# Electrophysiological investigations of peripheral nerves and muscles: a method for looking at cell dysfunction in the critically ill patients

**DOI:** 10.1186/s13054-019-2331-y

**Published:** 2019-01-29

**Authors:** Nicola Latronico, Oliver Friedrich

**Affiliations:** 1 Department of Medical and Surgical Specialties, Radiological Sciences and Public Health, University of Brescia, Brescia, Italy; 2 Department of Anesthesia, Intensive Care and Emergency, Spedali Civili University Hospital, Piazzale Ospedali Civili, 1, 25123 Brescia, Italy; 3 Institute of Medical Biotechnology, Department of Chemical and Biological Engineering, Friedrich-Alexander-University Erlangen-Nuernberg, Erlangen, Germany; 4 School of Medical Sciences, University of New South Wales, Sydney, Australia; 5 Victor Chang Cardiac Research Institute, Sydney, Australia

**Keywords:** Muscle weakness, Polyneuropathy, Myopathy, Organ dysfunction, Mitochondrial dysfunction, Energy metabolism

Resting trans-membrane potential difference (E_m_) of skeletal muscle is correlated to the energy status of the organism: the more severe the illness, the lower the E_m_. In 1971, Cunningham demonstrated this association with severely debilitating medical conditions, showing an increase in intracellular sodium concentration possibly due to a “generalized cellular abnormality” [1]. The study posed the basis for considering local (muscle) bio-electrical events generated by excitable tissues as indicative of the well-being of the entire organism. In 1995, Leijten showed that patients with electrophysiological signs of polyneuropathy had increased intensive care unit (ICU) mortality, more prolonged rehabilitation, and persistent 1-year motor handicap than those without [2]. In 1996, Latronico demonstrated normal nerve histology, despite electrophysiological findings of axonal neuropathy, in biopsies taken in the early stage of acute disease. In late biopsies, however, axonal nerve degeneration was evident [2]. This generated the hypothesis that functional (electrical) impairment may precede structural (histologic) changes and that electrophysiological study (EPS) might be used to look indirectly but non-invasively at cell functioning. During sepsis, a prototypical low-energy hyper-catabolic state, the nerves were trying to maintain their structure and survive by reducing or abolishing the function, a phenomenon easily documented by EPS. If sepsis persisted, the energy supply and/or use might not be restored and the histologic alterations would eventually ensue. According to this theory of the bioenergetic failure, “stunned but still living peripheral nerves and muscles may serve as a sentinel for the development of multiple organ dysfunction syndrome” [3]. In 1999, Hotchkiss described a similar divergence between in vivo clinical evidence of organ failure and *post-mortem* histologic absence of extensive organ damage sufficient to explain the morbidity and mortality of sepsis [4]. They also hypothesized that in situations of energy failure the cells may revert to a low energy state, a “hibernation” of the cell, to avoid cell death. The theory received support from two multi-center clinical studies, CRIMYNE [5] and CRIMYNE-2 [6], showing that the peroneal nerve, a long lower limb motor nerve, was the most commonly affected nerve. The axons are devoid of the machinery for biosynthetic processes, and all axonal components are synthesized in the cell body. Their anterograde transportation to the nerve terminal requires considerable energy expenditure and may fail if the nerve does not receive adequate nourishment [5].

**Fig. 1:**
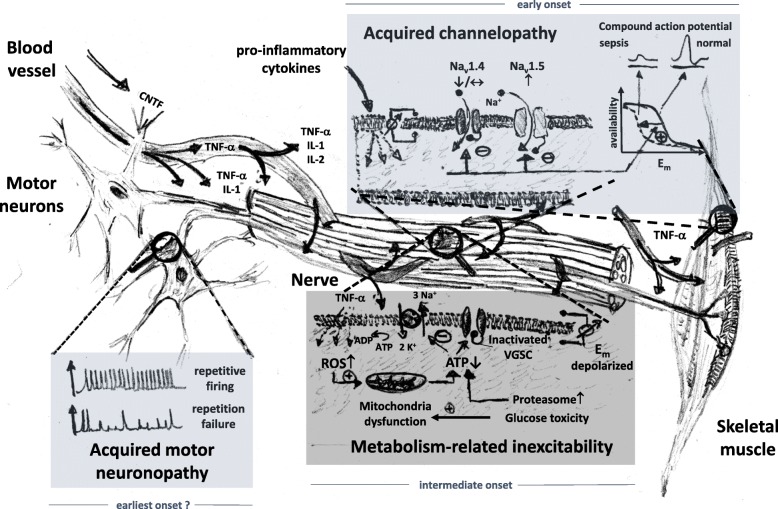
Mechanisms leading to neuromuscular inexcitability during early to intermediate phases of sepsis and critical illness. The very early phases of systemic inflammation are reflected by motor neuron repetitive firing failure followed by development of an acquired channelopathy of voltage-gated Na^+^-channels (VGSC) in peripheral nerves and muscles. In the course of critical illness, metabolism-related inexcitability adds another level through ATP depletion. At later stage, structural changes prevail (not shown). Reversibility declines with each added layer of mechanism. PKC, protein kinase C; CNTF, ciliary neurotrophic factor; ROS, reactive oxygen species

A reversible neuropathy was exactly matched in a rat sepsis model, with decreased action potential amplitudes of dorsal root axons by day 3 of sepsis with normal nerve morphology [7]. At this time point, there was no major resting membrane depolarization, nor decrease in membrane input resistance that could explain the reduced availability of voltage-gated Na^+^-channels (VGSC) for action potential transmission. Also, electrolytes were unaltered. Instead, a hyperpolarizing shift in Na^+^-channel inactivation was found as the predominant factor underlying the reversible nerve inexcitability [7]. This acquired VGCS channelopathy seems one of the earliest consequences of critical illness [7, 8]. Even preceding this electrical failure in axons may be the ability for coordinated repetitive firing within the motor neurons in the early phases of sepsis where nerve conduction still appears normal. Unstable motor neuron spikes during maintained direct current stimulation of spinal cord motor neurons in septic rats prevented a steady force production in muscle [9]. Decreased VGSC availability in skeletal muscles during experimental sepsis is attributed to upregulated expression of Na_v_1.5 isoforms (normally expressed during early maturation) over the adult Na_v_1.4 isoform [7]. Na_v_1.5 have a more negative half-inactivation potential compared to Na_v_1.4; thereby, the availability of activatable VGSC at normal E_m_ is already markedly reduced and the rheobase action potential is small and eventually fades. It has been speculated that this differential isoform expression represents a universal reaction of excitable organs to systemic inflammation [7]. The mechanism by which VGSC availability can also be reduced is through posttranslational modifications, e.g., phosphorylation. The VGSC α-subunit has several intracellular phosphorylation sites to modify Na^+^ ion gating as well as channel inactivation. Intriguingly, very fast phosphorylation of VGSCs (~minutes) was shown by exposure to pro-inflammatory cytokines (e.g., TNF-α, IL-2, CNTF ciliary neurotrophic factor), producing a maximum inhibition of 75% of Na^+^ currents through channel phosphorylation and hyperpolarizing shift of steady-state inactivation. This was abolished by protein kinase C blockade and also almost completely reversible after cytokine washout. This links the severity of neuropathy (and myopathy) in critical illness to the flush of pro-inflammatory cytokines, which, over time, crosses towards bioenergetics failure and, ultimately, structural damage. Inflammation, hypoxia, and ischemia increase local nitric oxide and reactive oxygen species production that lead to mitochondrial dysfunction and ATP depletion in nerve axons [8]. ATPase run-down results in depolarized E_m_ with persistent Na^+^ influx through nerve Na_v_1.6 and intracellular Ca^2+^ overload (reverse Na/Ca-exchanger activity) [8]. Cytokine-dependent activation of ATP-consuming proteolytic pathways worsens the situation and, in time, drives structural axonopathy, which is also promoted by glucose toxicity. These complex series of events, which are summarized in Fig. 1, find their clinical counterpart in the systemic inflammation and critical illness with artificial ventilation and mechanical silencing that ultimately lead to structurally-related organ failure, i.e., in nerves with structural axonopathies and in muscle with preferential myosin loss and muscle necrosis (reviewed in [10]).

Several clinical studies have shown an association of EPS alterations with clinical severity, most notably studies on intensive insulin treatment in surgical and medical ICU patients (reviewed in) [11], as well as an association with morbidity [12], and with hospital [13] and 1-year mortality [14]. Most recently, Kelmenson and colleagues have shown that patients with abnormal EPS had significantly fewer 28-day ICU-free days, a worse discharge disposition and higher mortality in the ICU and in the hospital when compared to patients with normal EPS [15]. So, in the last half century, a considerable amount of evidence has demonstrated that reduced bodily energy production, as it can be inferred from altered EPS, can be associated with disease severity and can be predictive of ominous prognoses. The time has come that we, as a clinical and scientific community devoted to unraveling the complex pathophysiology and timely treatment of multiple organ failure, would consider a wider adoption of EPS into the daily clinical practice.
